# Prevalence of Child Fever, Acute Respiratory Infection, and Diarrhea and Their Risk Factors in Sierra Leone

**DOI:** 10.3390/life14111493

**Published:** 2024-11-16

**Authors:** Hana Kim, Yan Jin

**Affiliations:** 1 Department of Global Development and Entrepreneurship, Graduate School of Global Development and Entrepreneurship, Handong Global University, Pohang 37554, Republic of Korea; 2 Department of Microbiology, Dongguk University College of Medicine, Gyeongju 38066, Republic of Korea

**Keywords:** fever, acute respiratory infection, diarrhea, Sierra Leone, demographic and health survey

## Abstract

Sierra Leone has the fourth-highest child mortality rate in Sub-Saharan Africa. This retrospective study aimed to estimate the nationwide prevalence of fever, acute respiratory infection (ARI), and diarrhea in children under 5 years in Sierra Leone, and to identify the risk factors associated with these diseases. We extracted patient information from the 2019 Sierra Leone Demographic and Health Survey data. Data were analyzed using multivariate logistic regression. In total, 8659, 8652, and 8650 children were included in the analyses of fever, ARI, and diarrhea, respectively. The analysis revealed that the nationwide prevalence rates of fever, ARI, and diarrhea were 16.8%, 14.2%, and 7.2%, respectively. Children aged 12–23 months were found to be the most susceptible. Compared to children consuming unimproved water, the highest prevalence rates of fever, ARI, and diarrhea were observed among children residing in households with tube wells or boreholes. The adjusted odds ratio for diarrhea prevalence among children drinking water from household tube wells or boreholes was 1.47 (95% confidence interval: 1.17–1.84, *p* < 0.001). This study has several limitations, including recall bias due to parental reporting. We suspect that the diseases may be associated with potential water contamination in tube wells or boreholes. We recommend national-level periodic inspections of water quality and community-level education on water supply management.

## 1. Introduction

Since the Sierra Leone government launched the Free Health Care Initiative (FHCI) to reduce the mortality rate of infants and children under 5 years in 2010 [1], the prevalence of fever, acute respiratory infection (ARI), and diarrhea has decreased by 27%, 25%, and 18%, respectively, as measured in 2017 [2]. However, the child mortality rate in Sierra Leone remained high at 101 per 1000 live births in 2022, ranking it fifth in Sub-Saharan Africa, following Niger, Nigeria, Somalia, and Chad [3]. The leading causes of child mortality in Sierra Leone are malaria (38%), pneumonia (7%), and diarrhea (8%) [4].

Access to healthcare in rural Sierra Leone is hindered by long travel distances, systemic corruption, and inadequate infrastructure and services [5]. The global health community has made significant efforts to address these challenges. For example, the International Organization for Migration and the Afro-European Medical and Research Network launched a Mobile Health Clinic campaign that operated in rural areas for approximately two years starting in 2017 [6]. However, this mobile clinic faced limitations in terms of space for equipment and patient handling, and it struggled to recruit healthcare personnel. Improving social determinants of health is considered the most effective strategy for reducing child mortality [7], with additional measures including the promotion of women’s education [8]. Globally, women with higher education levels are better informed about nutrition and healthcare, tend to have fewer children, and marry later in life [9]. In developing countries such as Malawi, Uganda, Ghana, and Bangladesh, higher educational attainment among mothers is associated with decreased child morbidity and mortality [10,11,12]. Children of self-determined mothers generally enjoy better health [9]. According to the Malaria Behavior Survey of 2019, women who demonstrated a positive attitude toward prompt treatment were 3.6 times more likely than others to seek immediate treatment for a child with fever at a public health center [13].

This study aimed to compare and analyze the demographic, socioeconomic, living environment, and community-dwelling characteristics of children under 5 years with and without fever, ARI, and diarrhea in Sierra Leone. It also sought to identify the risk factors associated with these conditions within the 2 weeks preceding the survey. Research exploring the prevalence of and risk factors for these diseases, based on a nationwide survey in Sierra Leone, is scarce. Therefore, evaluating factors related to fever, pneumonia, and diarrhea using the most recent data from the 2019 Sierra Leone Demographic and Health Survey (SLDHS) may help to bridge the research gap with other countries and identify the major determinants associated with these diseases in children under 5 years in Sierra Leone. This study aimed to identify strategies for reducing morbidity and mortality associated with these three diseases in children under 5 years in Sierra Leone following the 2014 Ebola epidemic.

## 2. Materials and Methods

### 2.1. Study Context

The Republic of Sierra Leone, located on the Western Atlantic coast of Africa and bordered by Guinea and Liberia, is a presidential republic with a population of approximately 8.60 million [14]. The country endured an 11-year civil war driven by conflicts over diamond rights, which resulted in Sierra Leone having the world’s highest proportion of people with disabilities and the lowest life expectancy, largely due to the forced recruitment of juvenile soldiers [15]. As of 2022, the life expectancy in Sierra Leone remains among the lowest globally at 60 years [16]. In response to high morbidity and mortality rates related to maternal, newborn, and child health, the Sierra Leone government launched the FHCI in April 2010. This initiative ensured the availability of essential services for mothers and children under 5 years and improved equity in treatment [17]. However, the mortality rate for children under 5 years rose from 139.2 per 1000 in 2014 to 140.2 per 1000 in 2015, as the FHCI was suspended from May 2014 to November 2015 due to the Ebola outbreak [3]. Although mortality rates decreased following the end of the Ebola outbreak in 2016, 40% of adolescent girls dropped out of school during the crisis, and rates of early marriage and adolescent births increased [18]. The Sierra Leone government enforced school bans on pregnant girls and adolescent mothers until 2020 [19]. Additionally, the government is investing in women’s education to enhance education systems, health practices, and learning conditions, enabling girls to resume formal education after childbirth [20].

### 2.2. Study Design and Data Source

This study was a nationwide cross-sectional analysis of data from the Sierra Leone Demographic and Health Survey (SLDHS). The survey, conducted in 2019 by the Sierra Leone Statistical Office, received funding from several organizations, including the United States International Development Agency, the Department of International Development, the United Nations Population Fund, the World Health Organization (WHO), and the World Bank. The SLDHS has been carried out every 5 years since 2008. The 2019 iteration adhered to the Demographic and Health Survey (DHS)-7 program standard. The questionnaire covered various aspects concerning households, mothers, and children under 5 years. Data collection took place from 15 May to 31 August 2019, with fieldwork beginning on 15 May 2019.

SLDHS 2019 used stratified two-stage cluster sampling. The study utilized stratified sampling by categorizing 16 districts across five regions as either urban or rural. The sampling framework was derived from the 2015 Sierra Leone Population and Housing Census conducted by the Sierra Leone Statistical Office. In the first stage, 578 enumeration areas (EAs; 214 urban and 364 rural) were selected in proportion to the population size of each EA. Subsequently, households within each EA were chosen using systematic sampling to enhance the representativeness of the data from both rural and urban areas across Sierra Leone. To gather statistical information that accurately represents the entire country, the number of samples surveyed in each region needed to closely match the actual distribution across the country while being proportionate to the population size of the region. However, if the population in some areas is sparse, sufficient samples may not be included; thus, the sample weight was adjusted in proportion to the area’s size.

The survey included women aged 15–49 years. Interviews were conducted with either permanent residents of selected households or visitors who had stayed in the household the night before the survey.

### 2.3. Study Model

**Figure 1:**
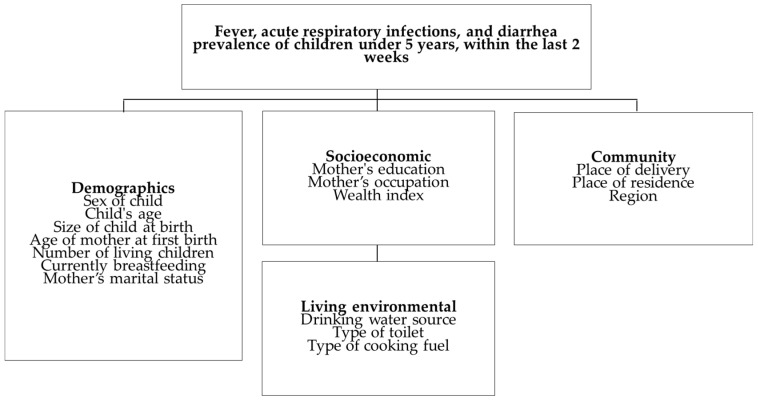
Conceptual framework.

The dependent variable in this study was the incidence of fever, ARI, and diarrhea among children under 5 years over the past two weeks. The independent variables were categorized into key determinants at the individual, household, and community levels, following Mosley and Chen’s conceptual framework [21], which facilitates the evaluation of overall health impacts on our development strategy and identifies several variables as impact factors [22] (Figure 1).

### 2.4. Study Participants

**Figure 2:**
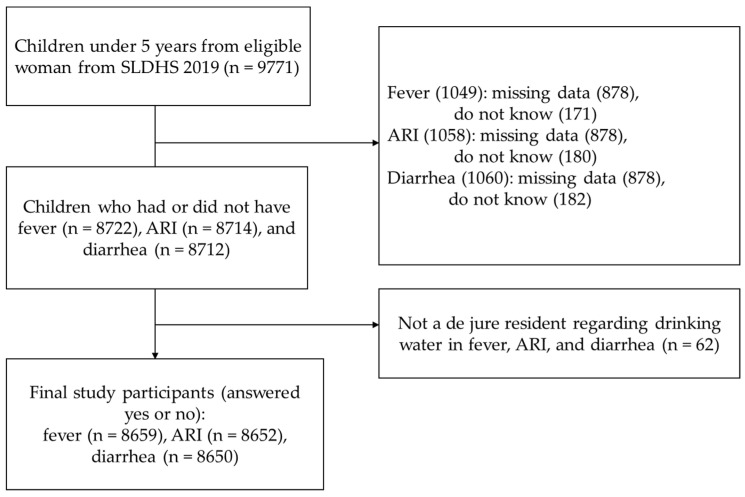
Selection of study participants. ARI, acute respiratory infection.

The participants in this study were children under 5 years, born to women aged 15–49 years in Sierra Leone. A total of 16,099 women were selected for individual interviews, with 15,574 successfully completed interviews, yielding a response rate of 97%. Out of the interviewed women, 9771 had children under 5 years. Two weeks prior to the survey, respondents who answered “do not know” regarding fever (171), ARI (180), or diarrhea (182), as well as those who did not respond (878), were excluded from the study. The number of children under 5 years affected by these three conditions was 8722 for fever, 8714 for ARI, and 8712 for diarrhea. Additionally, 62 respondents who were not legal residents (non-de jure residents) were excluded from answering questions related to drinking water sources and diarrhea. Among the final study participants, 8659 were analyzed for fever, 8652 for ARI, and 8650 for diarrhea (Figure 2).

### 2.5. Statistical Analyses

The results of frequency analysis, chi-square tests, and logistic regression analysis were conducted using SPSS version 23 (IBM Corp., Armonk, NY, USA). We estimated the nationwide weighted prevalence of fever, ARI, and diarrhea. To confirm the independence of variables from socioeconomic characteristics, correlation analysis was performed. Variables with a correlation coefficient of ≥0.6 were excluded from the multivariate logistic regression analysis. A multivariate logistic regression analysis, employing the backward elimination method, was used to calculate adjusted odds ratios (AORs) with 95% confidence intervals (95% CIs). This analysis identified variables with a significance level of <5%. Additionally, the Hosmer and Lemeshow goodness-of-fit test was conducted to assess the suitability of the model.

#### 2.5.1. Dependent Variables

The incidences of fever, ARI, and diarrhea in children under 5 years within the 2 weeks prior to the survey served as dependent variables. A child was considered to have fever, ARI, or diarrhea if, during the 2 weeks before the survey, they experienced fever, reported coughing or shortness of breath with pain, or had loose or wet stools more than three times a day, respectively. Respondents answered “yes”, “no”, or “do not know” to questions about these conditions. To analyze the variables associated with these three diseases, responses of “do not know” or those with “missing values” were excluded from the study. Consequently, the dependent variables were categorized based solely on “yes” or “no” answers.

#### 2.5.2. Independent Variables

The independent variables were categorized into demographic, socioeconomic, living environmental, and community characteristics of children under 5 years. The counts of children with fever, ARI, and diarrhea were 8659, 8652, and 8650, respectively. The characteristics of the study participants, according to factors related to fever, ARI, and diarrhea, are presented in Figure 1.

The demographic characteristics analyzed included the sex and age of the child (in months), birth size, the mother’s age at first birth, the number of living children, the current breastfeeding status in the household, and the mother’s marital status. Children were categorized by sex and grouped by age into three ranges: 0–11 months, 12–23 months, and 24–59 months, following classifications used in prior studies to differentiate between infants and toddlers [23,24]. To assess prevalence based on birth size, children were classified as small, average, or large. The average age of mothers at the time of their first childbirth was 19.24 years. The ages were grouped into three categories: 12–17 years, 18–25 years, and over 26 years. The number of living children in a household was categorized as one, two, three, four, or more. The current breastfeeding status was simply classified as either “yes” or “no.” The marital status of the mothers was categorized as never married, married, living with a partner, or separated, divorced, or bereaved.

The socioeconomic characteristics analyzed included the mother’s education level, occupation, and household wealth index. The education level of mothers was categorized into four groups: no education, primary, secondary, and higher education. Similarly, mothers’ occupations were classified as not working, working in service, agriculture and fishery, skilled/unskilled sectors, and others. Economic status was divided into five quintiles: poorest, poor, middle, rich, and richest.

The characteristics of the living environment included sources of drinking water, types of toilets, and types of cooking fuel. Drinking water sources were categorized as either improved or unimproved according to the Joint Monitoring Program for Water Supply and Sanitation standards. Unimproved water sources encompassed unprotected wells, springs, and surface water, while improved sources included piped water, tube wells or boreholes, protected dug wells and springs, rainwater, packaged water, delivery water, and water kiosks. Notably, the Millennium Development Goals consider packaged and delivered water as unimproved, whereas the Sustainable Development Goals classify them as improved drinking water [25,26,27]. Toilets were categorized as either improved toilets that were safely managed or unimproved toilets. Improved toilets primarily consisted of flush and pit toilets, while those lacking facilities were deemed unimproved. Cooking fuel was classified into clean and unclean fuels based on WHO standards [27]. 

Community characteristics included both the type of residence and the geographical region. Residences were categorized as either urban or rural. Regions were classified into five categories: western, eastern, northern, northwestern, and southern.

### 2.6. Ethical Considerations

The DHS protocol underwent review by both the Sierra Leone Ethics and Scientific Review Board and the International Classification of Functioning, Disability, and Health Agency Review Board. Prior consent was obtained from all participants before conducting the survey. All DHS data were analyzed anonymously. Access was granted to published public data [28].

## 3. Results

Table 1, Table 2, Table 3 and Table 4 display various variables related to disease prevalence according to participant characteristics. The proportion of male participants was slightly higher than that of females. The analysis revealed that of the mothers surveyed, 3162 (36.5%) were under 17 years old, 4894 (56.5%) were between 18 and 25 years old, and 604 (7.0%) were over 26 years old at the time of their first childbirth. The average number of living children per household was 3.1. Regarding marital status, 971 mothers (11.2%) were unmarried, 7360 (85%) were married or living with a partner, and 329 (3.8%) were separated due to divorce or bereavement (Table 1).

The educational background of the mothers varied: 4715 (54.4%) had no formal education, 1251 (14.4%) had a primary education, 2451 (28.3%) had a secondary education, and 243 (2.8%) had higher education. Additionally, employment status among the mothers showed that 1646 (19.0%) were not working, 2217 (25.6%) were employed in the service sector, 4337 (50.1%) worked in agriculture and fisheries, and 455 (5.3%) were in skilled or unskilled jobs. A total of 3949 children (45.6%) were categorized in the poorest wealth index groups (Table 2).

Regarding sources of drinking water, 3255 individuals (37.6%) relied on unimproved sources, while 1486 (17.2%) had access to piped water. Additionally, 1641 (19%) used tube wells or boreholes, 1843 (21.3%) utilized dug wells and springs, 136 (1.6%) collected rainwater, and 298 (3.4%) depended on packaged or delivered water. In terms of sanitation, 4352 participants (50.3%) had access to improved toilets, but only 19 (0.2%) used clean cooking fuel (Table 3).

As pertains to the place of delivery, 1375 children (15.9%) were born at their own or someone else’s house, 7271 (84%) were delivered at private or public healthcare facilities, and 13 (0.2%) were born at other locations. In total, 3063 (35.4%) resided in urban areas, while 5596 (64.6%) lived in rural areas. The western region had the smallest population, with 1588 residents (18.3%; Table 4).

**Figure 3:**
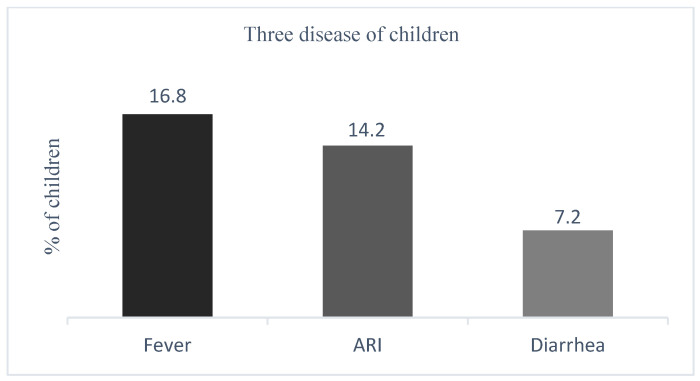
Weighted prevalence of fever, ARI, and diarrhea among children under 5 years.

The nationwide prevalence of fever, ARI, and diarrhea among children under 5 years within the 2 weeks prior to the survey was 16.8%, 14.2%, and 7.2%, respectively (Figure 3).

Table 5 presents the outcomes of the multivariate logistic regression analysis examining the association between fever, ARI, or diarrhea and various demographic, socioeconomic, living environment, and community characteristics. These factors are crucial in understanding the prevalence of fever, ARI, and diarrhea.

(1) Factors associated with the prevalence of fever:

Children’s age; size at birth; the age of the mother at first childbirth; current breastfeeding status; the mother’s marital status, education level and occupation; drinking water source; type of toilet used; and region were associated with the occurrence of fever. Children aged 12–23 months exhibited a 1.46-times higher prevalence of fever (AOR, 1.46; 95% CI, 1.22–1.74) compared to those aged 0–11 months. The prevalence of fever was 0.8 times lower in breastfed children (AOR, 0.8; 95% CI, 0.70–0.92) than in those who were not breastfed. Children of married and separated mothers had a 1.24 (AOR, 1.24; 95% CI, 1.01–1.51) and 1.5 times (AOR, 1.5; 95% CI, 1.07–2.09) higher prevalence of fever, respectively, compared to children of unmarried mothers. Children whose mothers received a primary education had a 1.26 times higher prevalence of fever (AOR, 1.26; 95% CI, 1.07–1.48) than those whose mothers had no formal education. Additionally, children of mothers employed in the service sector experienced a 1.35-times higher prevalence of fever (AOR, 1.35; 95% CI, 1.13–1.62) compared to children of mothers who were not working.

Children using tube wells or boreholes as improved drinking water sources had a 1.57-times higher prevalence of fever compared to those using unimproved sources (AOR, 1.57; 95% CI, 1.34–1.84). Children with access to improved toilets had a 35% lower likelihood of having fever than those with unimproved toilets (AOR, 0.65; 95% CI, 0.57–0.75). Compared to children living in the western region, those in the northern region had 41% lower odds of having fever (AOR, 0.59; 95% CI, 0.40–0.63).

(2) Factors associated with the prevalence of ARI:

Children’s age, size at birth, number of living children, mother’s education level and occupation, drinking water source, and region were associated with the occurrence of ARI in children under 5 years in Sierra Leone. Children aged 12–23 months had a 1.24-times higher prevalence of ARI (AOR, 1.24; 95% CI, 1.03–1.47) compared to those aged 0–11 months, while those aged 24–59 months had 20% lower odds (AOR, 0.8; 95% CI, 0.69–0.94). The odds of ARI were 26% lower (AOR, 0.74; 95% CI, 0.61–0.88) in average-sized and 29% lower (AOR, 0.71; 95% CI, 0.59–0.86) in large-sized children at birth compared to small-sized children. Households with four or more children had 18% lower odds of ARI (AOR, 0.82; 95% CI, 0.68–0.99) than those with only one child. Children of mothers employed in the service sector had a 1.39-times higher prevalence of ARI (AOR, 1.39; 95% CI, 1.16–1.67) compared to children of non-working mothers. Children using piped water and those using tube wells or boreholes had a 1.39-times (AOR, 1.39; 95% CI, 1.13–1.70) and 1.48-times (AOR, 1.48; 95% CI, 1.25–1.77) higher prevalence of ARI, respectively, compared to those using unimproved drinking water. Children living in the western region exhibited the highest prevalence of ARI. Those in the eastern and northern regions were 43% (AOR, 0.57; 95% CI, 0.45–0.72) and 28% (AOR, 0.72; 95% CI, 0.57–0.91) less likely to suffer from ARI compared to those in the western region.

(3) Factors associated with the prevalence of diarrhea:

Children’s age, size at birth, age of the mother at first childbirth, mother’s marital status and education level, drinking water source, and the region were associated with the occurrence of diarrhea in children under 5 years in Sierra Leone. Children aged 12–23 months experienced a 1.73-times higher prevalence of diarrhea (AOR, 1.73; 95% CI, 1.35–2.20) compared to those aged 0–11 months. The odds of diarrhea were 25% lower in average-sized (AOR, 0.75; 95% CI, 0.59–0.95) and 29% lower in large-sized children (AOR, 0.71; 95% CI, 0.55–0.91) at birth than in small-sized children. Children of mothers aged 18–25 years and those over 26 years at first childbirth had 22% (AOR, 0.78; 95% CI, 0.66–0.93) and 31% (AOR, 0.69; 95% CI, 0.47–1.00) lower odds of having diarrhea, respectively, compared to children of mothers aged under 17 years at first birth. Children with separated mothers had a 2.39-times higher prevalence of diarrhea (AOR, 2.39; 95% CI, 1.55–3.70) than those whose mothers had never married. Children of mothers with only a primary education had a 1.3-times higher prevalence of diarrhea (AOR, 1.3; 95% CI, 1.04–1.62) compared to those whose mothers had no formal education. Children using tube wells or boreholes for drinking water had a 1.47-times higher prevalence of diarrhea (AOR, 1.47; 95% CI, 1.17–1.84) than those using unimproved sources. Compared to children living in the western region, which had the highest prevalence of diarrhea, those in the northern and southern regions were 55% (AOR, 0.45; 95% CI, 0.32–0.62) and 33% (AOR, 0.67; 95% CI, 0.49–0.90) less likely to suffer from diarrhea, respectively.

We further analyzed the results presented in Table 6 to understand the relation between drinking water sources and infection prevalence.

Association between mother’s education level and water, sanitation and hygiene:

Mothers with a primary, secondary, and higher education were 1.3, 1.57, and 2.76 times more likely, respectively, to use improved drinking water sources than mothers without any formal education. Additionally, mothers with a formal education were more likely to use improved toilets compared to those without a formal education (Table 6). 

Association between household wealth index and water sanitation and hygiene:

The likelihood of using improved water sources was higher across various household income levels, with poor, middle-income, rich, and richest households being 1.79, 2.16, 2.39, and 3.82 times more likely, respectively, to use such sources compared to the poorest households. Additionally, all other households were more likely to use improved toilets than poor households (Table 6). 

## 4. Discussion

This study determined that the nationwide prevalence rates of fever, ARI, and diarrhea in children under 5 years in Sierra Leone were 16.8, 14.2, and 7.2%, respectively.

The key findings of this study are as follows: children whose mothers had completed primary education were more likely to experience fever, ARI, and diarrhea compared to those whose mothers had no education. Additionally, mothers with a primary education were more inclined to use tap or tube water compared to those without any formal education. Furthermore, individuals using tap or tube water exhibited higher odds of suffering from fever, ARI, and diarrhea than those who did not.

The association between the type of water source and the prevalence of diarrhea was unexpected. Children from households where mothers had higher educational levels and wealth indices were more likely to use improved water sources and toilets. The prevalence of fever, ARI, and diarrhea was lower among children who used improved toilets compared to their counterparts. However, the incidence of fever, ARI, and diarrhea was highest among children who consumed water from tube wells or boreholes, compared to those drinking from unimproved sources. This suggests potential contamination in tube wells or boreholes [29,30,31,32]. For instance, in a recent study conducted in Freetown, Sierra Leone, 25% of standpipes were found to be contaminated at least an intermediate risk level of *E. coli* [32]. In Bangladesh, a higher detection rate of *Escherichia coli* has been reported in unsealed tube wells [33].

In Sierra Leone, only 58% of the population has access to improved drinking water supplies, and substantial proportion of these sources are contaminated due to a lack of WASH awareness [29,30]. Additionally, there is a prevalent distrust among Sierra Leoneans towards tube wells, leading to a preference for packaged water [31]. The contamination of water in Sierra Leone is attributed to several factors, including heavy rains and flooding during the summer rainy season, sewage leakage into boreholes, inadequate sanitation practices, and inefficient waste management [34,35]. Therefore, it is crucial to implement rigorous facility management and water testing to mitigate potential sources of contamination such as flooding. Moreover, educating the public on proper water management practices is essential to address these public health concerns.

Children aged 12–23 months are the most likely to experience fever, ARI, and diarrhea. This increased susceptibility is attributed to their exposure to various pathogens as they begin to crawl and increase their indoor and outdoor activities after their first year [36]. In Sierra Leone, 99.8% of the population relies on biomass fuels such as wood and charcoal. With population growth, the demand for these fuels is expected to rise, leading to increased air pollution from forest burning and fuel use, which poses significant health risks to women and children [37]. However, the prevalence of ARI could decrease with the adoption of cleaner fuel alternatives [38].

In addition, as mothers cease breastfeeding, the disease protection provided by breast milk diminishes. Additionally, as children start consuming solid foods, they face increased risks of ingesting contaminated food or handling food with unclean hands [39]. A 2013 qualitative study on the causes of diarrhea among children under 5 years in rural Sierra Leone revealed that mothers struggle to control the activities of children older than 12 months. These activities include walking, running, playing in dirt, walking barefoot, and maintaining hand hygiene throughout the day [40,41]. Consequently, there is an urgent need for educational programs to help mothers recognize the causes of their children’s illnesses and manage their hygiene and nutrition effectively during this critical period of decreased immunity.

The study suggested that the children of adolescent mothers are more likely to develop diarrhea, possibly because adolescent mothers are more likely to give birth to underweight children who suffer from severe childhood diseases and mental and physical disabilities [42]. Underweight children face a mortality rate twice that of normal-weight infants within the first month of life and have a higher risk of developing acute diarrhea or pneumonia [43]. According to a 2022 study by the United Nations Children’s Fund, Sierra Leone had the highest rate of early marriages among women living in the poorest households, in rural areas, or those with only a primary education, with 3 out of 10 marrying before the age of 18. Although Sierra Leone’s free primary education policy, implemented in 2004, lowered entry barriers to primary education, it did not guarantee the quality of education [44,45]. Furthermore, since 2010, the Sierra Leone government has officially prohibited pregnant women from attending schools. This policy, coupled with school closures during the Ebola outbreak, has hindered female adolescents’ access to secondary and higher education and contributed to increased rates of early marriage in Sierra Leone [19].

In our study, children with low birth weights exhibited a higher prevalence of ARI and diarrhea compared to their normal-weight counterparts. Additionally, children whose mothers had only a primary education were more prone to developing fever and diarrhea than those whose mothers had no formal education. Further research is needed to explore the underlying causes of these findings.

Numerous studies have demonstrated a correlation between maternal education and child infection rates, particularly when comparing mothers with a secondary education to those with a lower level of education. In western Sierra Leone, women with secondary education exhibited a positive attitude and provided primary care to children with malaria [46]. Additionally, in Ghana, the prevalence of diarrhea among children of mothers with a secondary or higher education was lower than that among children of mothers without a formal education [11]. Furthermore, the mortality rate of children under 5 years is reduced by 61% in India and 43% in Nigeria, respectively, if all women complete secondary education [47].

In August 2018, the government of Sierra Leone initiated the Free Quality School Education policy, which abolished tuition fees in primary and secondary public schools to ensure access to quality education [48]. If these policies are rigorously enforced and remain unaffected by external circumstances, a significant improvement in child health can be anticipated, provided the quality of education is maintained.

Children whose mothers were separated from their spouses exhibited a higher likelihood of developing fever, ARI, and diarrhea compared to children of mothers who were never married. Women who experienced separation through divorce or bereavement faced ongoing stress due to financial challenges, loneliness, social stigma, and the responsibilities of single parenting, which in turn impacted their children’s well-being [49,50,51].

## 5. Limitations

Our study had several limitations. The DHS data lacked variables related to local cultural practices that could influence child morbidity. Additionally, the use of cross-sectional data restricted our ability to draw causal conclusions between the dependent and independent variables [28]. The prevalence of the three diseases was assessed based on mothers’ self-reports from the two weeks preceding the survey, potentially introducing recall bias [28]. The SLDHS employed the household wealth index as a surrogate for household wealth because the survey did not collect data on individual household income and expenditures. Furthermore, as the SLDHS 2019 survey was conducted during the rainy season (May–November), the findings might differ if collected during the dry season.

## 6. Conclusions

Using data from the national SLDHS 2019, we estimated the prevalence of fever, ARI, and diarrhea in children under 5 years across Sierra Leone, as well as the factors associated with these conditions. Children whose mothers had completed primary education exhibited higher odds of experiencing fever, ARI, and diarrhea compared to those whose mothers had no education. Additionally, mothers with a primary education were more likely to utilize tap or tube water than those without any formal education. Correspondingly, children consuming tap or tube water demonstrated increased odds of having fever, ARI, and diarrhea compared to those who did not. We strongly suspect that tap and tube well water sources in Sierra Leone may be contaminated. We recommend that policymakers develop comprehensive guidelines for the management of water sources, particularly improved sources such as tube wells and boreholes. It is crucial to conduct periodic water quality inspections at the national level and to provide community-level education on water supply management.

## Figures and Tables

**Table 1:** Disease prevalence by participant characteristics in Sierra Leone in 2019.

Variables		Fever (n = 8659)	ARI (n = 8652)	Diarrhea (n = 8650)
		N (%)	N (%)	N (%)
Sex of child	Male	4377 (50.5)	4375 (50.6)	4371 (50.5)
	Female	4282 (49.5)	4277 (49.4)	4279 (49.5)
Child’s age (months)	0–11	1941 (22.4)	1937 (22.4)	1941 (22.4)
	12–23	1813 (20.9)	1814 (21.0)	1812 (21.0)
	24–59	4905 (56.6)	4901 (56.6)	4896 (56.6)
Size of child at birth	Small	1100 (12.7)	1101 (12.7)	1100 (12.7)
	Average	4026 (46.5)	4017 (46.4)	4017 (46.4)
	Large	3335 (38.5)	3334 (38.5)	3336 (38.6)
	Do not know	199 (2.3)	200 (2.3)	197 (2.3)
Age of mother at first birth	≤17	3162 (36.5)	3152 (36.4)	3161 (36.5)
	18–25	4894 (56.5)	4896 (56.6)	4885 (56.5)
	≥26	604 (7.0)	604 (7.0)	603 (7.0)
Number of living children	1	1738 (20.1)	1738 (20.1)	1737 (20.1)
	2	2087 (24.1)	2086 (24.1)	2083 (24.1)
	3	1737 (20.1)	1733 (20.0)	1735 (20.1)
	≥4	3098 (35.8)	3094 (35.8)	3095 (35.8)
Currently breastfeeding	No	4271 (49.3)	4271 (49.4)	4261 (49.3)
	Yes	4388 (50.7)	4380 (50.6)	4389 (50.7)
Marital status	Never married	971 (11.2)	972 (11.2)	966 (11.2)
	Married	7360 (85.0)	7353 (85.0)	7357 (85.0)
	Separated	329 (3.8)	327 (3.8)	327 (3.8)

ARI, acute respiratory infection.

**Table 2:** Socioeconomic characteristics of study participants in Sierra Leone in 2019.

Variables		Fever (n = 8659)	ARI (n = 8652)	Diarrhea (n = 8650)
		N (%)	N (%)	N (%)
Mother’s education	No education	4715 (54.4)	4712 (54.5)	4714 (54.5)
	Primary	1251 (14.4)	1251 (14.5)	1252 (14.5)
	Secondary	2451 (28.3)	2446 (28.3)	2443 (28.2)
	Higher	243 (2.8)	243 (2.8)	242 (2.8)
Mother’s occupation	Not working	1646 (19.0)	1646 (19.0)	1643 (19.0)
	Service	2217 (25.6)	2216 (25.6)	2216 (25.6)
	Agricultural and fishery	4337 (50.1)	4333 (50.1)	4335 (50.1)
	Skilled/unskilled	455 (5.3)	454 (5.2)	453 (5.2)
	Other	4 (0.0)	4 (0.0)	4 (0.0)
Wealth index	Poorest	2021 (23.3)	2022 (23.4)	2017 (23.3)
	Poor	1928 (22.3)	1925 (22.2)	1929 (22.3)
	Middle	1764 (20.4)	1761 (20.4)	1763 (20.4)
	Rich	1606 (18.5)	1602 (18.5)	1603 (18.5)
	Richest	1341 (15.5)	1342 (15.5)	1338 (15.5)

ARI, acute respiratory infection.

**Table 3:** Living environment of study participants in Sierra Leone in 2019.

Variables		Fever (n = 8659)	ARI (n = 8652)	Diarrhea (n = 8650)
		N (%)	N (%)	N (%)
Drinking water source	Unimproved	3255 (37.6)	3254 (37.6)	3254 (37.6)
	Piped water	1486 (17.2)	1486 (17.2)	1483 (17.1)
	Tube well, borehole	1641 (19.0)	1637 (18.9)	1642 (19.0)
	Dug wells, springs	1843 (21.3)	1840 (21.3)	1837 (21.2)
	Rain water	136 (1.6)	136 (1.6)	135 (1.6)
	Packaged/delivered water	298 (3.4)	298 (3.4)	298 (3.4)
Type of toilet	Unimproved	4307 (49.7)	4305 (49.8)	4302 (49.7)
	Improved	4352 (50.3)	4347 (50.2)	4348 (50.3)
Type of cooking fuel	Unclean	8641 (99.8)	8633 (99.8)	8631 (99.8)
	Clean	19 (0.2)	19 (0.2)	19 (0.2)

ARI, acute respiratory infection.

**Table 4:** Community characteristics of study participants in Sierra Leone in 2019.

Variables		Fever (n = 8659)	ARI (n = 8652)	Diarrhea (n = 8650)
		N (%)	N (%)	N (%)
Place of delivery	Home	1375 (15.9)	1377 (15.9)	1376 (15.9)
	Health facility	7271 (84.0)	7262 (83.9)	7261 (83.9)
	Other	13 (0.2)	13 (0.2)	13 (0.2)
Type of residence	Urban	3063 (35.4)	3060 (35.4)	3056 (35.3)
	Rural	5596 (64.6)	5592 (64.6)	5594 (64.7)
Region	Western	1588 (18.3)	1590 (18.4)	1588 (18.4)
	Eastern	1854 (21.4)	1848 (21.4)	1847 (21.3)
	Northern	1746 (20.2)	1746 (20.2)	1745 (20.2)
	Northwestern	1626 (18.8)	1627 (18.8)	1626 (18.8)
	Southern	1845 (21.3)	1842 (21.3)	1844 (21.3)

ARI, acute respiratory infection.

**Table 5:** Multivariate logistic analyses of factors associated with fever, ARI, and diarrhea in children under 5 years in Sierra Leone in 2019.

Variable	Fever	*p*-Value	ARI	*p*-Value	Diarrhea	*p*-Value
	AOR (95% CI)		AOR (95% CI)		AOR (95% CI)	
Child age (months)						
0–11 ^a^	-		-		-	
12–23	1.46 (1.22, 1.74)	<0.001	1.24 (1.03, 1.47)	0.02	1.73 (1.35, 2.2)	<0.001
24–59	0.92 (0.77, 1.1)	0.34	0.80 (0.69, 0.94)	0.005	1.04 (0.84, 1.3)	0.72
Size of child at birth						
Small ^a^	-		-		-	
Average	0.90 (0.76, 1.08)	0.26	0.74 (0.61, 0.88)	0.001	0.75 (0.59, 0.95)	0.02
Large	1.03 (0.86, 1.23)	0.75	0.71 (0.59, 0.86)	<0.001	0.71 (0.55, 0.91)	0.01
Do not know	0.53 (0.32, 0.87)	0.01	0.35 (0.2, 0.64)	0.001	0.74 (0.41, 1.35)	0.33
Age of mother at first childbirth (years)						
≤17 ^a^	-				-	
18–25	0.92 (0.81, 1.04)	0.17			0.78 (0.66, 0.93)	0.01
≥26	0.80 (0.62, 1.03)	0.09			0.69 (0.47, 1)	0.04
Number of living children						
1 ^a^			-			
2			1.05 (0.88, 1.26)	0.57		
3			1.04 (0.86, 1.27)	0.67		
≥4			0.82 (0.68, 0.99)	0.04		
Currently breastfeeding						
No ^a^	-					
Yes	0.80 (0.7, 0.92)	0.002				
Current marital status						
Never married ^a^	-				-	
Married	1.24 (1.01, 1.51)	0.04			1.23 (0.92, 1.65)	0.17
Separated	1.5 (1.07, 2.09)	0.02			2.39 (1.55, 3.7)	<0.001
Mother’s education level						
No education ^a^	-		-		-	
Primary	1.26 (1.07, 1.48)	0.01	1.07 (0.89, 1.28)	0.46	1.3 (1.04, 1.62)	0.02
Secondary	0.97 (0.84, 1.13)	0.72	0.92 (0.78, 1.08)	0.29	0.82 (0.67, 1.02)	0.07
Higher	1.33 (0.91, 1.94)	0.14	0.84 (0.57, 1.26)	0.40	0.98 (0.58, 1.68)	0.95
Mother’s occupation						
Not working ^a^	-		-			
Service	1.35 (1.13, 1.62)	0.001	1.39 (1.16, 1.67)	<0.001		
Agricultural and fishery	1.04 (0.86, 1.25)	0.71	0.98 (0.8, 1.2)	0.87		
Skilled/unskilled	0.99 (0.73, 1.34)	0.95	1.24 (0.92, 1.67)	0.15		
Other	1.45 (0.11, 19.34)	0.78	5.35 (0.66, 43.73)	0.12		
Drinking water source						
Unimproved water ^a^	-		-		-	
Piped water	1.09 (0.9, 1.32)	0.39	1.39 (1.13, 1.7)	0.002	1.12 (0.86, 1.47)	0.34
Tube well or borehole	1.57 (1.34, 1.84)	<0.001	1.48 (1.25, 1.77)	<0.001	1.47 (1.17, 1.84)	0.001
Dug well and spring water	1.11 (0.93, 1.33)	0.25	1.09 (0.9, 1.33)	0.37	1.1 (0.86, 1.4)	0.46
Rain water	1.15 (0.71, 1.85)	0.57	1.25 (0.76, 2.05)	0.38	1.19 (0.58, 2.42)	0.63
Packaged/delivered water	0.8 (0.54, 1.19)	0.27	1.1 (0.76, 1.6)	0.62	1.3 (0.79, 2.14)	0.29
Type of toilet						
Unimproved ^a^	-					
Improved	0.65 (0.57, 0.75)	<0.001				
Region						
Western ^a^	-		-		-	
Eastern	1.12 (0.91, 1.37)	0.29	0.57 (0.45, 0.72)	<0.001	1.03 (0.79, 1.36)	0.82
Northern	0.5 (0.4, 0.63)	<0.001	0.72 (0.57, 0.91)	0.006	0.45 (0.32, 0.62	<0.001
Northwestern	0.93 (0.75, 1.16)	0.51	0.88 (0.7, 1.12)	0.31	1.14 (0.85, 1.52)	0.38
Southern	0.99 (0.8, 1.23)	0.94	0.79 (0.63, 1.01)	0.06	0.67 (0.49, 0.9)	0.01
Fever	Nagelkerke R^2^ = 0.047, Hosmer and Lemeshow Test: χ^2^ = 5.565 (*p* = 0.696)					
ARI	Nagelkerke R^2^ = 0.036, Hosmer and Lemeshow Test: χ^2^ = 20,804 (*p* = 0.008)					
Diarrhea	Nagelkerke R^2^ = 0.041, Hosmer and Lemeshow Test: χ^2^ = 6.761 (*p* = 0.563)					

ARI, acute respiratory infection; AOR, adjusted odds ratio: all the variables in this table were controlled for adjusted analysis. 95% CI: 95% confidence interval; ^a^, reference category.

**Table 6:** Relationship of WASH with the mother’s education level and the household’s wealth index.

Variables		Improved Water Source (n = 9822)		Improved Toilet (n = 9822)	
		OR (95% CI)	*p*-Value	OR (95% CI)	*p*-Value
Mother’s education	No education ^a^	-		-	
	Primary	1.30 (1.15, 1.46)	<0.001	1.22 (1.09, 1.38)	0.001
	Secondary	1.57 (2.5, 3.1)	<0.001	2.94 (2.67, 3.24)	<0.001
	Higher	2.76 (6.54, 16.47)	<0.001	13.86 (9.21, 20.88)	<0.001
Wealth index	Poorest ^a^	-		-	
	Poor	1.79 (1.6, 2.02)	<0.001	3.47 (2.99, 4.02)	<0.001
	Middle	2.16 (1.92, 2.43)	<0.001	7.27 (6.28, 8.42)	<0.001
	Rich	2.39 (2.11, 2.71)	<0.001	31.57 (26.64, 37.42)	<0.001
	Richest	3.82 (3.3, 4.42)	<0.001	128.97 (97.27, 170.99)	<0.001

CI: confidence interval; ^a^, reference category; WASH, water, sanitation, and hygiene status.

## Data Availability

The datasets generated and/or analyzed during the current study are available in the DHS program repository, [https://dhsprogram.com/data/available-datasets.cfm (accessed on 21 January 2022)].
